# *ARHGEF39*, a Gene Implicated in Developmental Language Disorder, Activates RHOA and Is Involved in Cell De-Adhesion and Neural Progenitor Cell Proliferation

**DOI:** 10.3389/fnmol.2022.941494

**Published:** 2022-07-25

**Authors:** Midas Anijs, Paolo Devanna, Sonja C. Vernes

**Affiliations:** ^1^Neurogenetics of Vocal Communication Group, Max Planck Institute for Psycholinguistics, Nijmegen, Netherlands; ^2^School of Biology, The University of St Andrews, St Andrews, United Kingdom; ^3^Donders Institute for Brain, Cognition and Behaviour, Nijmegen, Netherlands

**Keywords:** *ARHGEF39*, Rho GTPases, RHOA, cell adhesion, scRNA-seq, neural progenitor cells (NPCs), cell division

## Abstract

*ARHGEF39* was previously implicated in developmental language disorder (DLD) *via* a functional polymorphism that can disrupt post-transcriptional regulation by microRNAs. ARHGEF39 is part of the family of Rho guanine nucleotide exchange factors (RhoGEFs) that activate small Rho GTPases to regulate a wide variety of cellular processes. However, little is known about the function of *ARHGEF39*, or how its function might contribute to neurodevelopment or related disorders. Here, we explore the molecular function of ARHGEF39 and show that it activates the Rho GTPase RHOA and that high ARHGEF39 expression in cell cultures leads to an increase of detached cells. To explore its role in neurodevelopment, we analyse published single cell RNA-sequencing data and demonstrate that *ARHGEF39* is a marker gene for proliferating neural progenitor cells and that it is co-expressed with genes involved in cell division. This suggests a role for *ARHGEF39* in neurogenesis in the developing brain. The co-expression of *ARHGEF39* with other RHOA-regulating genes supports RHOA as substrate of ARHGEF39 in neural cells, and the involvement of RHOA in neuropsychiatric disorders highlights a potential link between ARHGEF39 and neurodevelopment and disorder. Understanding the GTPase substrate, co-expression network, and processes downstream of *ARHGEF39* provide new avenues for exploring the mechanisms by which altered expression levels of ARHGEF39 may contribute to neurodevelopment and associated disorders.

## Introduction

Developmental speech and language disorders are highly heritable, with most cases showing complex multifactorial inheritance (Reader et al., 2014). This complex genetic aetiology makes the identification of risk genes challenging, but investigating the biological function of these genes offers an important gateway for understanding the biological basis of human speech and language (Deriziotis and Fisher, 2017). Previously, *ARHGEF39* was identified as a potential risk factor for a syndrome of unexplained language problems in children known as developmental language disorder (DLD) (Devanna et al., 2018). At the time of that study, this condition was labelled as specific language impairment, but DLD is now the recommended terminology (Bishop et al., 2017). *ARHGEF39* was implicated following a screen of non-coding 3′UTR sequences for variation that could disrupt microRNA (miR) binding sites in children with DLD (Devanna et al., 2018). A single nucleotide polymorphism in the *ARHGEF39* 3′UTR (rs72727021) was associated with a quantitative measure of language impairment (non-word repetition) and functional assays in cell models showed that the risk allele disrupted regulation of ARHGEF39 by miR-215. Expression quantitative trait loci data further indicated that the DLD-associated allele was associated with higher expression of ARHGEF39 in post-mortem human brain (Devanna et al., 2018). However, little is known about the biological function of *ARHGEF39*, the role of this gene in neurodevelopmental processes, or how variation in these processes may contribute to human language development or disorder.

*ARHGEF39* is one of 82 Rho guanine nucleotide exchange factors (RhoGEFs) in the human genome (Fort and Blangy, 2017). RhoGEFs initiate the activation of Rho GTPases by stimulating them to bind GTP instead of GDP (Etienne-Manneville and Hall, 2002). Rho GTPases are involved in every cellular process that requires cytoskeletal reorganisation (Etienne-Manneville and Hall, 2002; Rossman et al., 2005). The most extensively characterised Rho GTPases, CDC42, RAC, and RHO, stimulate the re-organisation of the cytoskeleton into distinct cellular structures upon activation: filopodia, lamellipodia, and focal adhesions, respectively (Nobes and Hall, 1995). In neurodevelopment, CDC42, RAC, and RHO each have specific contributions to various processes, such as neurite outgrowth, axon pathfinding, and dendritic spine development *via* their effects on the cytoskeleton, membrane trafficking and microtubule dynamics (Govek et al., 2005). Each RhoGEF controls these processes by activating one or more of the Rho GTPases, meaning that the RhoGEFs substrate specificity is deterministic of its biological function. Aberrant Rho GTPase signalling is implicated in multiple neurodevelopmental disorders. Genes in Rho GTPase signalling pathways are enriched in rare CNVs associated with autism as well as in GWAS hits for schizophrenia and bipolar disorder (Pinto et al., 2010; Zhao et al., 2015). Specific RhoGEFs have been implicated in neurodevelopmental disorders, such as language impairment (*ARHGEF19*), intellectual disability (*ARHGEF6*, *ARHGEF2*), and moderate intellectual disability with speech delay (*ARHGEF9*) (Kutsche et al., 2000; de Ligt et al., 2012; Nudel et al., 2014; Ravindran et al., 2017). These finding highlight the potential importance of Rho GTPase signalling and its regulation in neurodevelopmental disorders.

Most studies on *ARHGEF39* have investigated its role in cancer, while the developmental and neurobiological roles of *ARHGEF39* have received limited attention, leaving open questions about its contribution to neurodevelopmental phenotypes and disorders. In hepatocellular and lung cancer, increased expression of *ARHGEF39* has been reported as a prognostic factor for tumour size and patient survival (Zhou et al., 2018; Gao and Jia, 2019; Xu et al., 2019; Cooke et al., 2021). Furthermore, overexpression of *ARHGEF39* leads to increased proliferation, migration, and invasion of cancer cells (Wang et al., 2012, 2018; Zhou et al., 2018; Cooke et al., 2021). These studies demonstrate a molecular link between *ARHGEF39* and cancer cell phenotypes. However, to understand the role of ARHGEF39 in neurodevelopment or related disorders, we need to understand its molecular function, and study its expression patterns and potential molecular interactions in relevant models. In this study, we aim to do this by investigating the substrate specificity of *ARHGEF39* and consequences of its overexpression. We also utilise existing single cell RNA-seq datasets to determine the expression of ARHGEF39 in the developing brain and uncover neurodevelopmental processes implicated in its function.

## Materials and Methods

### Cell Culture and Transfection

Biosensor assays were performed in human HEK293FT cells. Cells were obtained from ThermoFisher and were routinely screened for mycoplasma contamination. All experiments were carried out using cells grown in Dulbecco’s modified Eagle’s media (Invitrogen) supplemented with 10% foetal bovine serum (Sigma-Aldrich) and 2 mM penicillin/streptomycin. Cells were maintained at 37°C in the presence of 5% CO_2_. Transfections were performed using GeneJuice (Novagen) following the manufacturer’s instructions.

### Expression Vectors

Several expression vectors were used for these experiments. A vector expressing ARHGEF39 (pcDNA3.1-ARHGEF39) was obtained from NovoPro (catalogue # 718357). Plasmids encoding second generation FRET-based Rho GTPase biosensor were obtained from Addgene: pTriEx4-Rac1-2G (#66110), pTriExRhoA2G (#40176), pTriEx4-Cdc42-2G (#68814) (Fritz et al., 2013, 2015; Martin et al., 2016). ARHGDIA was PCR amplified from cDNA from SH-SY5Y cells with the following primers: ARHGDIA_*Hin*dIII_Fw TTACTAAGCTTATGGCTG AGCAGGAGCCCACAG and ARHGDIA_*Kpn*I_Rv TTACTGG TACCGTCCTTCCAGTCCTTCTTGATG. The PCR product was cloned into the pcDNA3.1 expression vector using *Hin*dIII and *Kpn*I restriction sites to create pcDNA3.1-ARHGDIA. The sequence was confirmed by Sanger sequencing.

### FRET-Based Rho GTPase Biosensor Assay

HEK293FT cells were seeded in poly-D-lysine-coated glass-bottom 96-well plates at 20,000 cells per well and were allowed to adhere overnight. Cells were transfected with 20 ng of pTriEx4-Rac1-2G, pTriExRhoA2G, or pTriEx4-Cdc42-2G and 10 ng of pcDNA3.1-ARHGDIA and 0, 20, 60, or 100 ng of pcDNA3.1-ARHGEF39. The minimum amount of ARHGDIA that was needed to increase the dynamic range of the essay was determined in a dose-response experiment (Supplementary Figure 1). pcDNA3.1-empty was used as filler to keep total DNA content constant across conditions. Medium was changed to FluoroBrite DMEM (ThermoFisher) with 10% foetal bovine serum 1 h before reading the plate. Images of cells were captured 48 h after transfection.

Biosensor assays were performed on a Tecan Infinite M200 PRO plate reader with a temperature-controlled incubation chamber at 37°C and 5% CO_2_. Excitation wavelength was set at 453 nm and an emission scan was read from the bottom of each well between 487 and 600 nm with a 1 nm step size. For each experiment (*n* = 3), three wells per Rho GTPase:ARHGEF39 ratio were measured. Background fluorescence was measured in nine untransfected wells. Average background values were subtracted from the raw intensity values and spectra were normalised by area in a| e UV-Vis-IR Spectral Software (version 2.2^1^). Subsequently, the ratio between 528 nm and 492 nm ratio was calculated to determine Rho GTPase activation. Significant differences between groups were calculated using an ANOVA test followed by *post hoc* Tukey HSD test.

### Cell Adhesion

HEK293FT cells were seeded in 12-wells plates at 100,000 cells per well and were allowed to adhere overnight. Cells were transfected with 500 ng pcDNA3.1-ARHGEF39 or pcDNA3.1-empty. Cells were counted 48 h after transfection. Total culture medium was removed, centrifuged at 200xg for 3 min and resuspended in 100 ul of Dulbecco’s PBS (Sigma) to count the number of floating cells. Attached cells were detached from the plate with 0.25% trypsin-EDTA (Invitrogen) and resuspended in 1 ml of Dulbecco’s PBS after centrifugation (Sigma). Cells were stained with 0.4% Trypan Blue (BioRad) and counted with the TC20 automated cell counter (BioRad) according to the manufacturer’s instructions. Significant differences in total cell counts and viability percentages were calculated with a two-sided *t*-test. Viability percentages were arcsine transformed before statistical testing.

### Analysis of *ARHGEF39* in scRNA-Seq Data

Expression matrix and meta file of Loo et al. (2019); Polioudakis et al. (2019), and Fan et al. (2020) were downloaded from https://github.com/jeremymsimon/MouseCortex, http://solo.bmap.ucla.edu/shiny/webapp/, and Gene Expression Omnibus (GSE120046), respectively. These datasets were processed using Seurat v3.2.2 (Butler et al., 2018). Clusters were selected if ARHGEF39 expression value was larger than 0 in at least 10% of cells. For each of the selected clusters, cells were grouped in ARHGEF39-positive and ARHGEF39-negative cells for a differential gene expression analysis. The sizes of ARHGEF39-positive groups can be found in Supplementary Table 1. The smallest group was 70 ARHGEF39-positive cells for Loo_SVZ3. Differential gene expression analysis was performed with edgeR v3.28.1 (Robinson et al., 2010), using genewise negative binomial general linear models (glmFit) and likelihood ratio tests for the model (glmLRT). We used a cut-off value of FDR-corrected *p*-value < 0.01. GO enrichment analysis was performed on the basis of these DEGs and marker genes for radial glia, cluster 2 (E14.5) (Loo et al., 2019) and cycling progenitors (G2/M phase) (Polioudakis et al., 2019) by Metascape (Zhou et al., 2019). Metascape was set to use gene sets from Gene Ontology and Reactome with default parameters. The union of all genes expressed in at least 10% of a cluster in the Loo et al., Zhou et al., and Polioudakis et al. datasets (9360) was used as list of background genes. Overlap between DEG lists was statistically assessed with Fisher’s exact tests using the R package GeneOverlap v3.15.

## Results

### ARHGEF39 Activates RHOA GTPase

**FIGURE 1:**
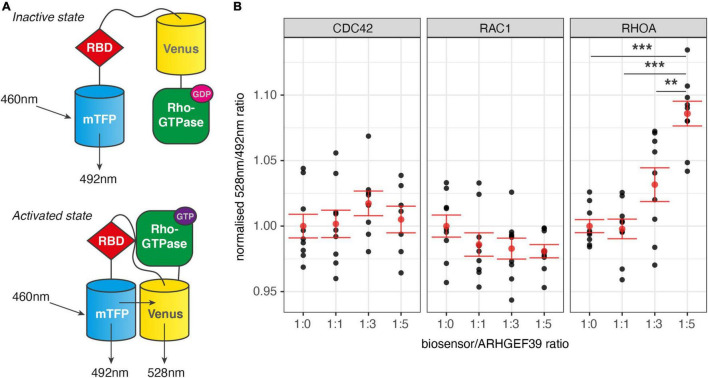
**(A)** Diagram of FRET-based Rho GTPase activity biosensor. If the Rho GTPase is an inactive GDP-bound state only a cyan fluorescent protein (mTFP) is excited. When the Rho GTPase is in an activated GTP-bound state, it is able to bind a Rho GTPase binding domain (RBD) in the biosensor. This induces a conformational change that enables a cyan fluorescent protein (mTFP) to excite a yellow fluorescent protein (Venus) through FRET. Activity is measured as the ratio between emissions at 528 nm and 492 nm. **(B)** Normalised ratio of emissions at 528 and 492 nm from Rho GTPase biosensors. Multiple ratios of biosensor vs. ARHGEF39 co-transfection were used: 1:0 (no ARHGEF39 control), 1:1, 1:3, and 1:5. Mean and standard error are indicated in red. *p*-values are determined with a Tukey HSD test after ANOVA. ***Indicates *p* < 0.001, **p < 0.01.

Given their distinct roles in remodelling the cytoskeleton, determining the Rho GTPase activation specificity of ARHGEF39 can provide a first clue in understanding its downstream functions. To test if CDC42, RAC1, or RHOA could be activated by ARHGEF39, we used second-generation genetically encoded Förster Resonance Energy Transfer (FRET) biosensors for each of the Rho GTPases of interest (CDC42, RAC1, and RHOA) (Fritz et al., 2013, 2015; Martin et al., 2016). These biosensor molecules contain the Rho GTPase of interest, a Rho GTPase binding domain (RBD) and two fluorophores. The excited donor fluorophore (mTFP) transfers energy to an acceptor fluorophore (Venus) that emits at a characteristic wavelength when the two fluorophores are brought in a close configuration as a result of the activated Rho GTPase binding the RBD (Figure 1A). We chose a widely used and highly tractable human cell line (HEK293FT cells) as a model for these tests as they focus on the general properties of the molecular interaction of ARHGEF39 with Rho GTPases rather than a cell type specific function. A plasmid overexpressing ARHGEF39 was co-transfected to HEK293FT cells in increasing quantities with a uniform amount of biosensor to test for Rho GTPase activation. Activation of CDC42 or RAC1 were not observed at any ratio. In contrast, RHOA showed significant activation when ARHGEF39 was overexpressed at a 1:5 transfection ratio to the biosensor (*p* = 0.000001) (Figure 1B).

### Overexpression of ARHGEF39 Increases the Amount of Cells in Suspension

**FIGURE 2:**
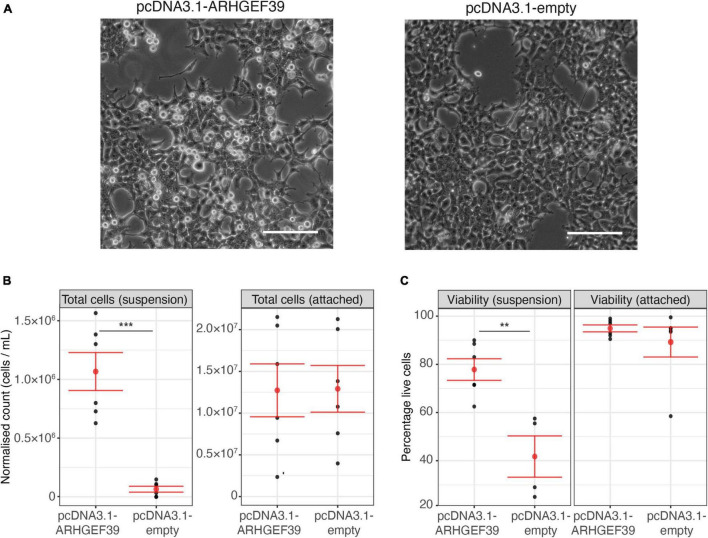
**(A)** Representative images of cells after transfection with an ARHGEF39 overexpressing vector (pcDNA3.1-ARHGEF39) or empty vector control (pcDNA3.1-empty). Scale bar indicates 500 um. **(B)** Counts of cells in suspension and attached cells 48 h after transfection with pcDNA3.1-ARHGEF39 and pcDNA3.1-empty. Concentrations for cells in suspension and attached cells were measured in 100 ul and 1 ml resuspension volumes, respectively (see Section “Materials and Methods”). Mean and standard error are indicated in red. **(C)** Viability of cells in suspension and attached cells 48 h after transfection with pcDNA3.1-ARHGEF39 and pcDNA3.1-empty. *p*-values are determined with two-sided *t*-test. ***Indicates *p* < 0.001, and **p < 0.01.

During our Rho GTPase activation experiments, we made an unexpected observation. In HEK293FT cell cultures that overexpressed ARHGEF39, more cells were observed floating in the culture media compared to cells that were transfected with an empty vector or EGFP control (Figure 2A). This increase of cells in suspension could have multiple explanations. Given that ARHGEF39 activates RHOA (Figure 1), this effect could be related to the established role of RHOA in the assembly of cell-matrix interaction *via* focal adhesions (Ridley and Hall, 1992; Nobes and Hall, 1995). Alternatively, it could indicate that high concentrations of ARHGEF39 are toxic and cause cell death driving more cells into suspension. To differentiate between these possibilities, we quantified the number of cells in suspension vs. attached cells and assessed cell viability to determine whether the cells had only detached or if they had also died.

The visual observation that an increased number of cells were in suspension after overexpression of ARHGEF39 was confirmed by automated cell counting (*p* = 0.0001) (Figure 2B). We also counted the attached cells and found no significant different between conditions (*p* = 0.96). Total cell counts were not significantly different between conditions (*p* = 0.59) as the increased number of cells in suspension made up a very small proportion of the total (∼0.5% of the control cells and ∼8.4% of the ARHGEF39 transfected cells). Trypan blue staining demonstrated that viability of the attached cells was high (>90%) in both conditions and no significant difference was observed (Figure 2C). Floating cells showed poor viability (∼42%) in the empty vector control condition for the small proportion of floating cells that could be found. In contrast, floating cells were largely viable (∼78%) in the ARHGEF39 overexpressing condition, which represents a substantial and significant increase compared to the control condition (*p* = 0.004). This suggests that high concentrations of ARHGEF39 are not toxic or causing cell death, rather, it is more likely that overexpression of ARHGEF39 leads to cell de-adhesion.

### *ARHGEF39* Is Expressed in Multiple Cell Types and Marks Out Neural Progenitor Cells

To understand its potential contributions to neurodevelopmental processes, we investigated ARHGEF39 expression in the developing brain using publicly available single cell RNA-sequencing (scRNA-seq) data. Large scRNA-seq datasets are available that describe the transcriptomes of individual cells. Unsupervised clustering methods applied to such data can group these cells based on similarity, after which the clusters are annotated for their properties such as cell type or state (Kiselev et al., 2019). This provides a powerful resource that we have leveraged to understand the cell types in which *ARHGEF39* is found in the brain and, by exploring the co-expressed genes, to suggest which pathways are active when *ARHGEF39* is expressed. We explored three recent scRNA-seq datasets that detail the development of mouse (Loo et al., 2019) and human neocortex (Polioudakis et al., 2019; Fan et al., 2020).

We first explored the marker genes of specific cell types that had been identified in the published cluster analyses. Here, a marker gene was defined as a gene that was significantly enriched in a cell type specific cluster during differential gene expression analysis when comparing the cluster with all other cells in the dataset. In the mouse and one of the human datasets, *ARHGEF39* was identified as a marker gene for clusters of neural progenitor cells (Loo et al., 2019; Polioudakis et al., 2019). In the embryonic mouse study, *Arhgef39* was classed as one of eight marker genes for one (cluster 2) of the four subpopulations of radial glia identified (Loo et al., 2019). In the mid-gestation human cortex study, *ARHGEF39* was among the 133 most significant differentially expressed genes for cycling progenitor cells in G2/M phase of the cell cycle (Polioudakis et al., 2019). These data suggest that *ARHGEF39* marks out specific populations of neural progenitor cells in mouse and human cortical development. Given the G2/M annotation of the human cluster, the function of ARHGEF39 may be related to this cell cycle phase.

Next, to identify all cell types in the developing brain in which ARHGEF39 could be found, we sought to identify the clusters in which *ARHGEF39* was reliably expressed in each dataset. Expression of ARHGEF39 in at least 10% of cells of a cluster was used as a threshold to identify positive cell types, and this identified nine ARHGEF39 positive clusters out of 71 clusters total (Supplementary Table 1). Neural progenitor cell clusters had the highest percentage of *ARHGEF39*-positive cells across all datasets supporting its status as a neural progenitor cell marker (Table 1). A role in neural development was further supported by bulk RNA-sequencing data from BrainSpan (Kang et al., 2011) and PsychENCODE (Li et al., 2018) which showed a prenatal enrichment for *ARHGEF39* (Supplementary Figure 2). In the PsychENCODE data, *ARHGEF39* was also assigned to co-expression module ME5, which is enriched for gene expression associated to radial glia and neural progenitor cells (Li et al., 2018). Other cell types that met the 10% threshold in the scRNA-seq datasets were cortex-adjacent ganglionic eminences in the developing mouse dataset (Loo et al., 2019), and cortical excitatory neurons and immune cells (containing microglia, macrophages, and T cells) in one of the developing human datasets (Fan et al., 2020). These data show that, while strongly enriched in neural progenitor cells, *ARHGEF39* is also present in multiple cell types in the developing brain.

**TABLE 1:** Cell clusters in which at least 10% of cells express ARHGEF39.

References	Species	Age	*ARHGEF39*^+^ clusters	# cells	% pos.
Loo et al. (2019)	Mouse	E14.5-birth	Radial glia, cluster 2 (E14.5)*	334	45.5%
			Subventricular zone, proliferating (E14.5)	315	22.2%
			Ganglionic eminences (Birth)	421	17.1%
			Ganglionic eminences (E14.5)	762	10.4%
Polioudakis et al. (2019)	Human	GW17-18	Cycling progenitors (G2/M phase)*	695	20.3%
Fan et al. (2020)	Human	GW7-28	Neural progenitor cells	1333	30.3%
			Early (GW7-9)	1392	22.0%
			Immune cells	511	15.3%
			Cortical excitatory neurons	2065	10.4%

*Clusters where ARHGEF39 was previously identified as a marker gene are denoted with an asterisk.*

### *ARHGEF39* Is Co-expressed With a Core Set of Genes in the Developing Brain

**FIGURE 3:**
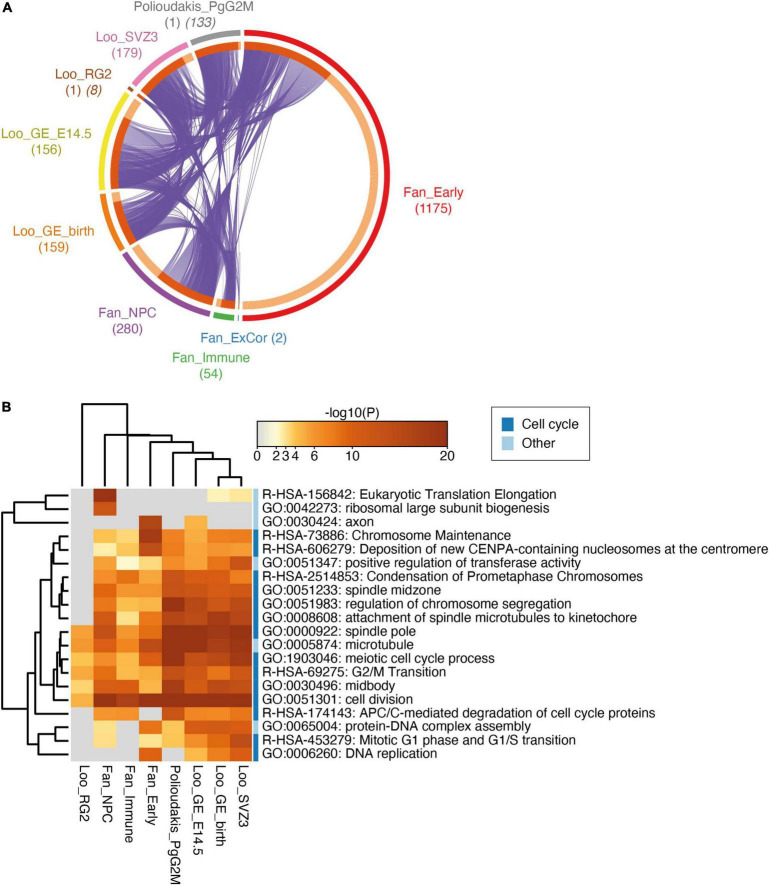
Differential gene expression analysis between ARHGEF39-positive and ARHGEF39-negative cells in ARHGEF39 expressing cell clusters. **(A)** Overlap between lists of DEGs. Purple curves link identical genes between lists. Genes that hit multiple lists are coloured in dark orange, and genes unique to a list are shown in light orange. Counts of DEGs are between brackets. For Loo_RG2 and Polioudakis_PgG2M, the number of cluster marker genes is added in oblique type. **(B)** Heatmap of top 20 enriched terms across lists of DEGs, coloured by *p*-values. Grey indicates a lack of significance. Dendrograms indicate similarity between terms (rows) and lists of DEGs (columns). Terms that are directly related to cell cycle processes are marked with a dark blue bar, other terms are marked with a light blue bar.

Next, we used these scRNA-seq data to investigate how *ARHGEF39* expression may affect the molecular state of the cells. Exploiting the cell-to-cell heterogeneity within clusters, we divided each cluster into *ARHGEF39*-positive and *ARHGEF39*-negative cells and performed differential gene expression analysis between these groups to find genes that correlate with *ARHGEF39* expression. The numbers of differentially expressed genes (DEGs) for each cluster are in Figure 3A. In the mouse radial glia cluster and human cycling progenitors cluster, where *ARHGEF39* was identified as a marker gene, *ARHGEF39* was the only DEG identified. The overall expression of *ARHGEF39*-positive cells and *ARHGEF39*-negative cells are very similar within these clusters, indicating that expression of *ARHGEF39* did not correlate with a specific cell type or cell state within these clusters. In the next step of our analysis, we took all other marker genes that were defined for radial glia 2 and cycling progenitors in G2/M phase, as ARHGEF39 likely correlates with a gene expression signature for these clusters as a whole (Loo et al., 2019; Polioudakis et al., 2019).

Strong overlap between the lists of DEGs and the lists of marker genes show that there is a core set of 46 genes that are co-expressed with ARHGEF39 (Figure 3A; Supplementary Figure 3 and Supplementary Table 3). Similarly to ARHGEF39, all these genes are in co-expression modules that are enriched for prenatal gene expression that is associated with radial glia and neural progenitor cells in the PsychENCODE human developmental transcriptome (Li et al., 2018). In a survey, 44 out of 46 core set genes are assigned to the same co-expression module as ARHGEF39, supporting the co-expression observed in single cell data (Supplementary Table 3). A large number of DEGs (1,175) were identified in the cluster of early cells from the Fan et al. (2020) dataset compared to the other clusters. The core set of *ARHGEF39* co-expressed genes is also detected in this cluster, but 79.4% of these DEGs did not overlap with other clusters. This can be explained by the type of annotation for this cluster. The cluster of early cells is not defined by cell type, but by gestational age (weeks 7–9) (Fan et al., 2020). As the early cell cluster contains a diversity of cell types, it also contains cell types that are ARHGEF39-negative. The non-overlapping DEGs are likely markers for these ARHGEF39-negative cell types that are included in the age-based early cell cluster but not in more cell type-based clusters. The core set of overlapping DEGs is present in all clusters and they likely correlate with a cell state in which ARHGEF39 is expressed (Figure 3A; Supplementary Figure 3).

### *ARHGEF39* Expression-Related Genes Are Involved in Cell Cycle Processes

To identify the biological processes that are associated with *ARHGEF39* expression we performed a gene ontology (GO) enrichment analysis on the genes within each of the clusters listed in Supplementary Table 2 (Figure 3B; Supplementary Table 4). Out of the top 20 enriched terms, 14 terms were directly related to cell cycle processes with three of the other terms (positive regulation of transferase activity, microtubule and protein-DNA complex assembly) still closely clustering to these 14 terms. A number of categories were significantly enriched across all clusters analysed, the most significant of which was “cell division” (adjusted *p*-value = 10^–60^). This is in line with *ARHGEF39* as a marker gene for cycling progenitors in G2/M phase. “G2/M transition” is the most widely and most significantly represented cell cycle phase in these gene lists (adjusted *p*-value = 10^–21^), However, the function of ARHGEF39 may not be limited to this phase as several of the involved genes overlap with other cell cycle phases, such as “mitotic G1 phase and G1/S transition” (adjusted *p*-value = 10^–12^) (Supplementary Table 4). DEGs from the cortical excitatory neuron cluster was not included in the GO enrichment analysis because this cluster produced too few DEGs (*N* = 2). A reason for this low number of DEGs might be that the majority of cells in this cluster of cortical excitatory neurons are post-mitotic. In this cluster, in addition to *ARHGEF39*, the only other DEG was *CDC25C (Cell Division Cycle 25C)*, which is an important cell cycle regulator involved in G2/M progression (Supplementary Table 2), which further supporting the link between *ARHGEF39* and cell cycle.

### *ARHGEF39* Is Co-expressed With RhoGEFs and RhoGAPs That Predominantly Regulate RHOA

Since Rho GTPase regulation is a dynamic and complex process orchestrated by multiple proteins, we also looked for RhoGEF and RhoGAP family members that may function in a regulatory manner with *ARHGEF39*. Several RhoGEF and RhoGAP family members (*N* = 17) were identified as DEGs (Supplementary Table 2) and these were all upregulated in *ARHGEF39*-positive cells (Supplementary Figure 2). A short list of RhoGEFs/RhoGAPs that were differentially expressed in at least two of the clusters was integrated with substrate specificity data from a comprehensive FRET-based Rho GTPase activity screen (Müller et al., 2020) to predict if they target the same substrates as ARHGEF39 (see Table 2). For the five RhoGEFs/RhoGAPs with significant results in the substrate specificity assays, four were shown to regulate RHOA, suggesting that RHOA is the most actively regulated Rho GTPase in cell states where ARHGEF39 is expressed. As such, these scRNA-seq data and previously published substrate specificity assays further support our findings from the biosensensor assays (Figure 1B) that RHOA is a substrate of ARHGEF39.

**TABLE 2:** RhoGEF and RhoGAP family members that are differentially expressed in at least two clusters Substrates are listed from a family-wide characterisation of substrate specificities of RhoGEFs and RhoGAPs in Müller et al. (2020).

Protein	Family	Substrate	Clusters	# of clusters
ARHGEF39	RhoGEF	n.s.	Fan_early, Fan_NPC, Polioudakis_PgG2M, Loo_GE_E14.5, Loo_GE_birth, Loo_SVZ3, Fan_Immune, Fan_ExCor, Loo_RG2	9
ECT2	RhoGEF	RHOA	Fan_early, Fan_NPC, Polioudakis_PgG2M, Loo_GE_E14.5, Loo_GE_birth, Loo_SVZ3	6
ARHGAP11A	RhoGAP	RHOA	Fan_early, Fan_NPC, Polioudakis_PgG2M, Loo_GE_E14.5, Loo_GE_birth, Loo_SVZ3	6
RACGAP1	RhoGAP	RAC1	Fan_early, Fan_NPC, Polioudakis_PgG2M, Loo_GE_E14.5, Loo_GE_birth, Loo_SVZ3	6
ARHGAP19	RhoGAP	RHOA	Fan_early, Fan_NPC Loo_GE_E14.5, Loo_GE_birth, Loo_SVZ3	5
DEPDC1	RhoGAP	n.s.	Fan_early, Fan_NPC, Polioudakis_PgG2M, Fan_Immune	4
ARHGAP11B	RhoGAP	RHOA, CDC42	Fan_early, Fan_NPC, Polioudakis_PgG2M	3
DEPDC1B	RhoGAP	n.s.	Fan_early, Fan_NPC, Polioudakis_PgG2M	3
OPHN1	RhoGAP	n.s.	Fan_early, Fan_NPC	2

*Not all RhoGEFs/RhoGAPs showed significant Rho GTPase activity upregulation or downregulation in this screen and these are indicated with n.s. (not significant).*

## Discussion

*ARHGEF39* was implicated in specific language impairment *via* a functional polymorphism in its 3′UTR that disrupted post-transcriptional expression regulation by microRNAs (Devanna et al., 2018). In this study, we identify RHOA as a substrate and downstream effector of ARHGEF39. We show that overexpression of ARHGEF39 disrupts cell adhesion. In the developing cortex, we report that *ARHGEF39* acts a marker for proliferating neural progenitor cells and is significantly co-expressed with genes involved in cell division. RHOA activity, cell de-adhesion, cell division, and neural progenitor cells present new avenues to explore how changes in *ARHGEF39* may contribute to neural development and to language disorder.

The direct activation of RHOA by ARHGEF39 is a novel finding. A family-wide screen of Rho GTPase activation by RhoGEFs using biosensors in HEK293T cells previously did not detect any significant activity of RHOA, RAC1, or CDC42 by ARHGEF39 (Müller et al., 2020). This previous study used a different ratio of ARHGEF39, inhibitor and biosensor. We optimised the sensitivity of this assay by determining the lowest effective dose of ARHGDIA to inhibit activation of the biosensor by endogenous Rho GTPases (Supplementary Figure 1). A significant effect on RHOA activation was only observed at the highest ratio (1:5) and not at the lower ratios (1:1 or 1:3). The increased sensitivity due to lower inhibition of the biosensors allowed us to uncover the effect of ARHGEF39 on RHOA using Rho GTPase biosensors. Another previous study exploring the interaction partners of ARHGEF39 identified RAC1, but not RHOA (or CDC42) in a pulldown assay from lung cancer cells overexpressing ARHGEF39 (Zhou et al., 2018). A recent study has added that ARHGEF39 is necessary for RAC1 activation during migration of lung cancer cells in response to growth factors (Cooke et al., 2021), but this study did not investigate any potential activation of RHOA. Our study showed that ARHGEF39 directly activates RHOA protein by using FRET-based Rho GTPase activity biosensors that measure activation in living cells. RAC1 activation was not detected, but this could be related to the lower propensity to migrate that HEK293FT cells have compared to lung cancer cells. It has been established that RHOA and RAC1 are mutually inhibitory Rho GTPases during cell migration, and that the activation of RAC1 is preceded by a brief peak in activation of RHOA at the leading edge that initiates protrusion (Machacek et al., 2009). This could point to a mechanism by which ARHGEF39 is indirectly involved in the activation of RAC1 by first activating RHOA in migrating cells, but this remains to be tested. The potential relevance of the ARHGEF39-RHOA pathway to neurodevelopment was highlighted by the co-expression of ARHGEF39 with cell division pathways and with other RHOA-regulating proteins in the developing brain (see further discussion, below).

Expression of ARHGEF39 has been shown to promote cell proliferation in cancer cells (Wang et al., 2012, 2018; Zhou et al., 2018). This role in cell proliferation is also supported for neural cells *via* our analyses of transcriptome-wide survey of *ARHGEF39*-associated gene expression across cell types in brain development. Combining the *ARHGEF39*-associated genes from multiple cell types presented a core network of genes that are involved in G2/M phase transition. During cell division, Rho GTPase activation is tightly regulated. RHOA and CDC42 are required for specific steps of remodelling the actin and microtubule cytoskeleton, whereas RAC1 must remain inactive during the entire process (Chircop, 2014). In particular, active RHOA is required during cell rounding, a process in which rigidity of the cell cortex increases and focal adhesions are disassembled (Maddox and Burridge, 2003). ARHGEF39 mediated activation of RHOA and subsequent increases in cell rounding could explain the increases in cell detachment observed when ARHGEF39 is overexpressed in HEK293FT cells. Although not yet directly tested, this theory is supported by high-resolution microscopy in COS-7 cells revealing that ARHGEF39 is localised to the plasma membrane and focal adhesions (Müller et al., 2020)–regions where RHOA activity is important for cell rounding (Maddox and Burridge, 2003). These findings from cell lines should be validated in neural cells to assess the importance of these processes in neurodevelopment. Notably, several genes in the co-expression network of *ARHGEF39* in neural cell types have functions related to mitotic cell rounding during cell division. ECT2 locally activates RHOA during G2 and M phase and is necessary for proper cell rounding and formation of the mitotic spindle and contractile ring (Tatsumoto et al., 1999; Yüce et al., 2005; Niiya et al., 2006; Matthews et al., 2012). RACGAP1 regulates cytokinesis by inactivating RAC1, recruitment of ECT2 and indirect activation of RHOA (Minoshima et al., 2003; Yüce et al., 2005; Bastos et al., 2012; Matthews et al., 2012; Zhang and Glotzer, 2015). DEPDC1B promotes disassembly of focal adhesions by displacing RHOA and makes RHOA available for other processes such as cortical stiffening (Marchesi et al., 2014). Future research will be needed to determine any direct or indirect interactions between ARHGEF39 and these co-expressed genes, and how they form molecular pathways that affect the function of neural cell types. Taken together, these data propose a mechanism by which ARHGEF39 and some of its co-expressed genes converge on RHOA activation and cell division to contribute to neurodevelopment.

We have shown that *ARHGEF39* is enriched in proliferating neural progenitor cells during cortical development. It would be of interest to study the molecular pathways and biological processes mediated by ARHGEF39 in these cell populations to understand its role in neurodevelopment, particularly since changes in Rho GTPase activation have emerged as a molecular hub in various neurodevelopmental disorders (Zhao et al., 2015; Zamboni et al., 2018). 16p11.2 deletion syndrome is a neurodevelopmental disorder that is characterised by a form of childhood apraxia of speech (Mei et al., 2018; Chung et al., 2021). Increased RHOA activity is a common feature of cellular and animal models for 16p11.2 deletion syndrome (Escamilla et al., 2017; Martin Lorenzo et al., 2021; Sundberg et al., 2021; Urresti et al., 2021). In human organoid models with 16p11.2 deletions, increased RHOA activation is observed alongside changes in proliferation, cell adhesion, and migration (Urresti et al., 2021). In the 16p11.2 deletion mouse model, altered cortical progenitor proliferation leads to an aberrant cortical cytoarchitecture that is characterised by a reduced number of upper layer neurons and increase in layer VI neurons (Pucilowska et al., 2015). Focal cortical abnormalities have further been observed in individuals with 16p11.2 deletions by MRI (Blackmon et al., 2018). It would be of interest to determine if mutations in *ARHGEF39* also lead to changes in cortical cytoarchitecture *via* the predicted changes in RHOA activity and cell division in mice or in humans.

From an evolutionary perspective, the increased proliferative capacity of human neural progenitor cells is considered important for the development of higher cognitive abilities, because of its role in the evolutionary expansion of the neocortex (Kalebic and Huttner, 2020). Human-specific gene *ARHGAP11B* is a prominent member in the network of *ARHGEF39*-associated genes, together with its ancient paralog ARHGAP11A. ARHGAP11B promotes the proliferation and delamination of radial glia cells, which may contribute to neocortical expansion (Florio et al., 2015). In order to explore the potential role of *ARHGEF39* in language-related neurodevelopment, it would be relevant to further study the cooperation of ARHGEF39 with ARHGAP11A and ARHGAP11B in neural progenitor cells, their role in cortical development, and how this may have changed over human evolution.

Together, these new insights on the molecular and cellular context of ARHGEF39 provide the bases for defining the role of ARHGEF39 in neurodevelopment. Future research into the effect of ARHGEF39 overexpression in neural progenitor cells on cell division, cell attachment and its co-expression network will be the next step for understanding the neurodevelopmental mechanisms that may be affected by *ARHGEF39* variants.

## Data Availability Statement

The original contributions presented in this study are included in the article/Supplementary Material, further inquiries can be directed to the corresponding author.

## Ethics Statement

Ethical review and approval was not required for the study in accordance with the local legislation and institutional requirements.

## Author Contributions

MA acquired and analysed the data and wrote the manuscript. All authors conceived the study, revised and edited the manuscript, and approved the submitted version.

## Conflict of Interest

The authors declare that the research was conducted in the absence of any commercial or financial relationships that could be construed as a potential conflict of interest.

## Publisher’s Note

All claims expressed in this article are solely those of the authors and do not necessarily represent those of their affiliated organizations, or those of the publisher, the editors and the reviewers. Any product that may be evaluated in this article, or claim that may be made by its manufacturer, is not guaranteed or endorsed by the publisher.

## Footnotes

1
http://www.fluortools.com/software/ae
