# Supplementary material for: ARHGEF39, a Gene Implicated in Developmental Language Disorder, Activates RHOA and Is Involved in Cell De-Adhesion and Neural Progenitor Cell Proliferation
Source: Front Mol Neurosci. 2022 Jul 25;15:941494. doi: 10.3389/fnmol.2022.941494 (PMC9359124; doi:10.3389/fnmol.2022.941494)
Supplement: Supplementary file 2 [file Data_Sheet_2.docx]

**Supplementary material**


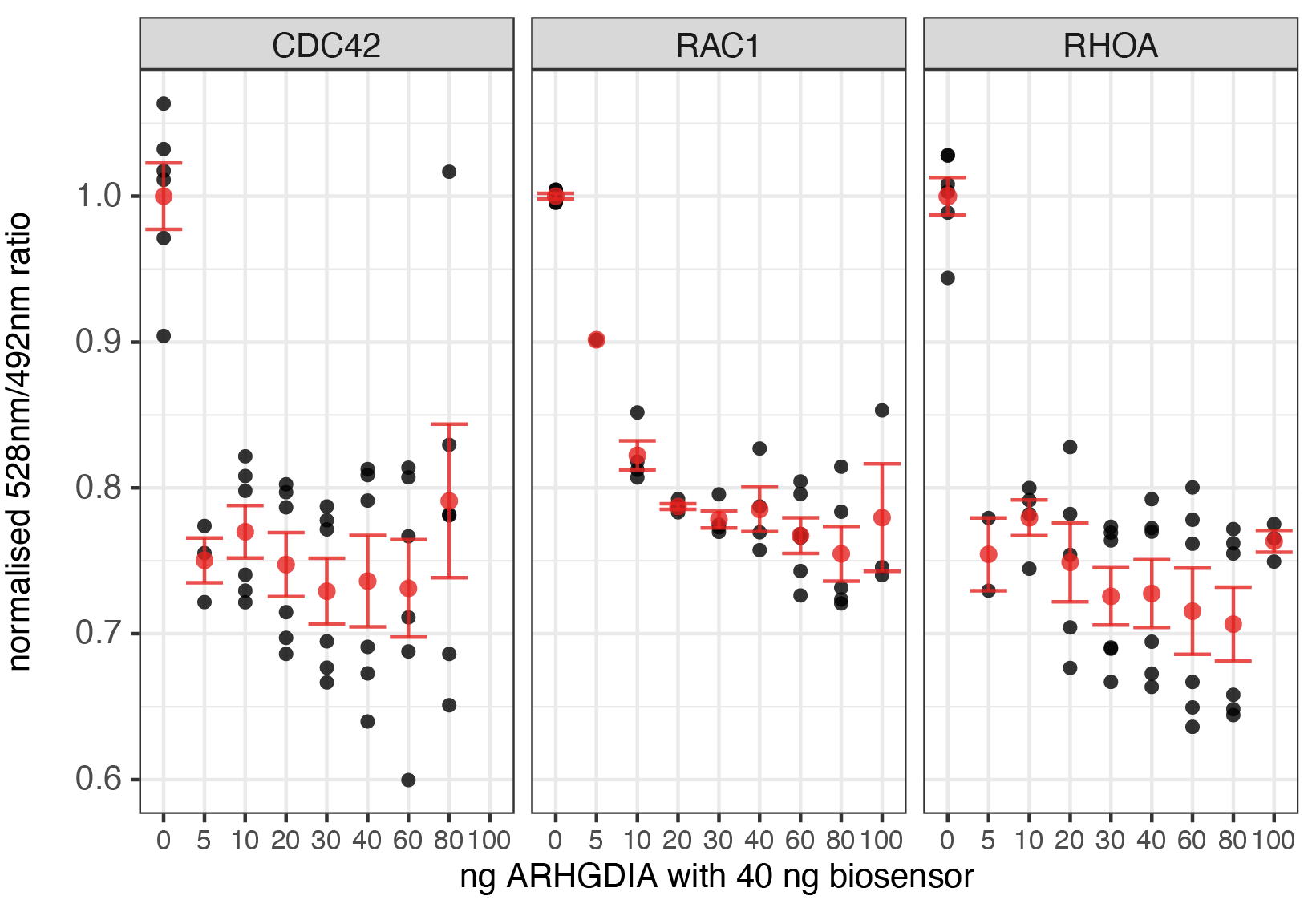


Figure S1. Titration of ARHGDIA for effective amount to increase dynamic range of FRET-based RhoGTPase biosensor assay


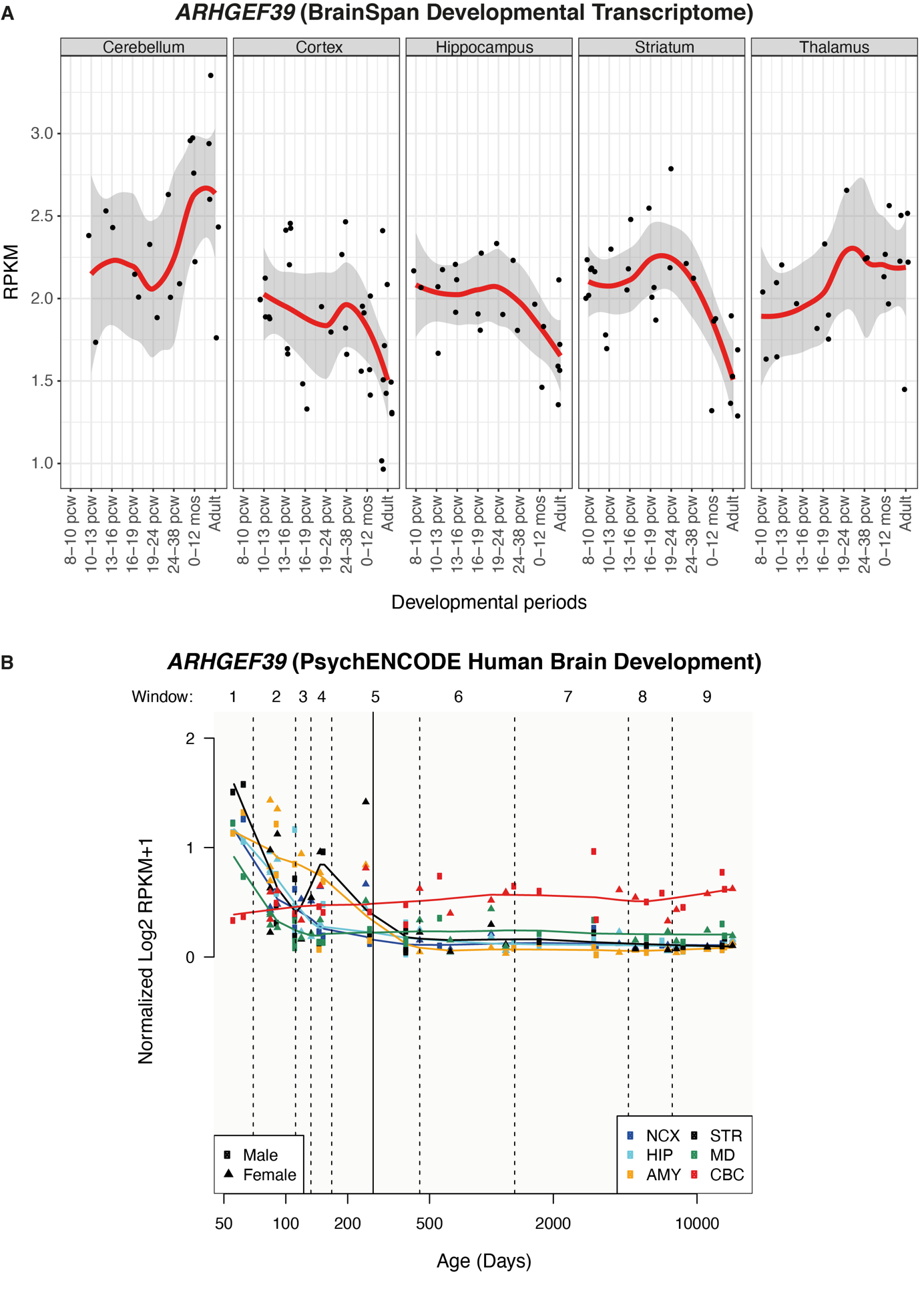


Figure S2: Developmental mRNA expression of ARHGEF39 in post-mortem samples from (A) BrainSpan Developmental Transcriptome and (B) PsychENCODE Human Brain Development.


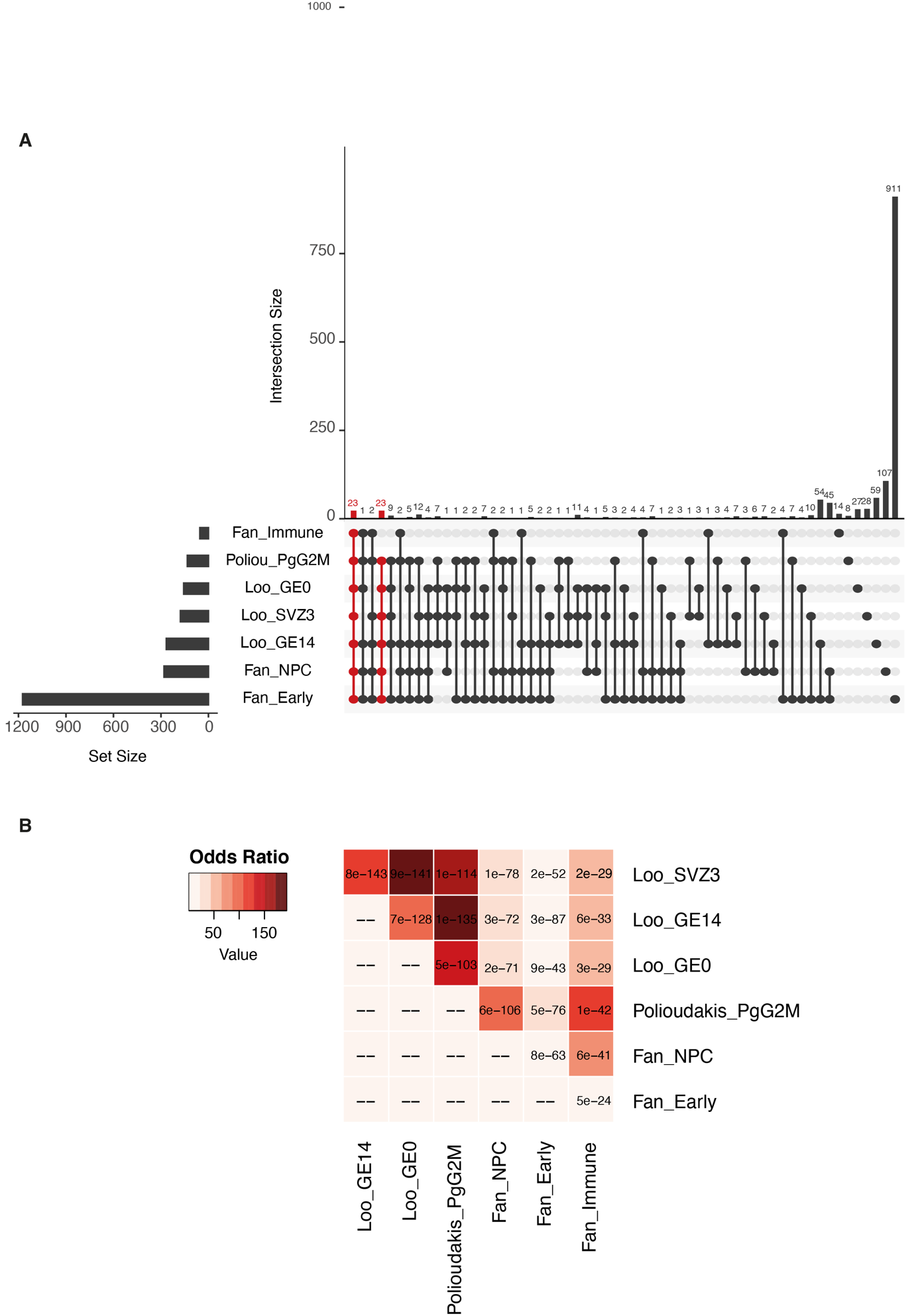


Figure S3. Overlap between lists of DEGs. (A) Upset plot of all intersections in ARHGEF39-associated gene lists. Core gene set is indicated in red. These are genes shared by Polioudakis_PgG2M, Loo_GE0, Loo_GE14, Loo_SVZ3, Fan_NPC and Fan_Early. (B) Gene overlap matrix. Odds ratios in shades of red indicate the strength of association between two gene lists. Fisher’s exact test p-values are superimposed on the grid.


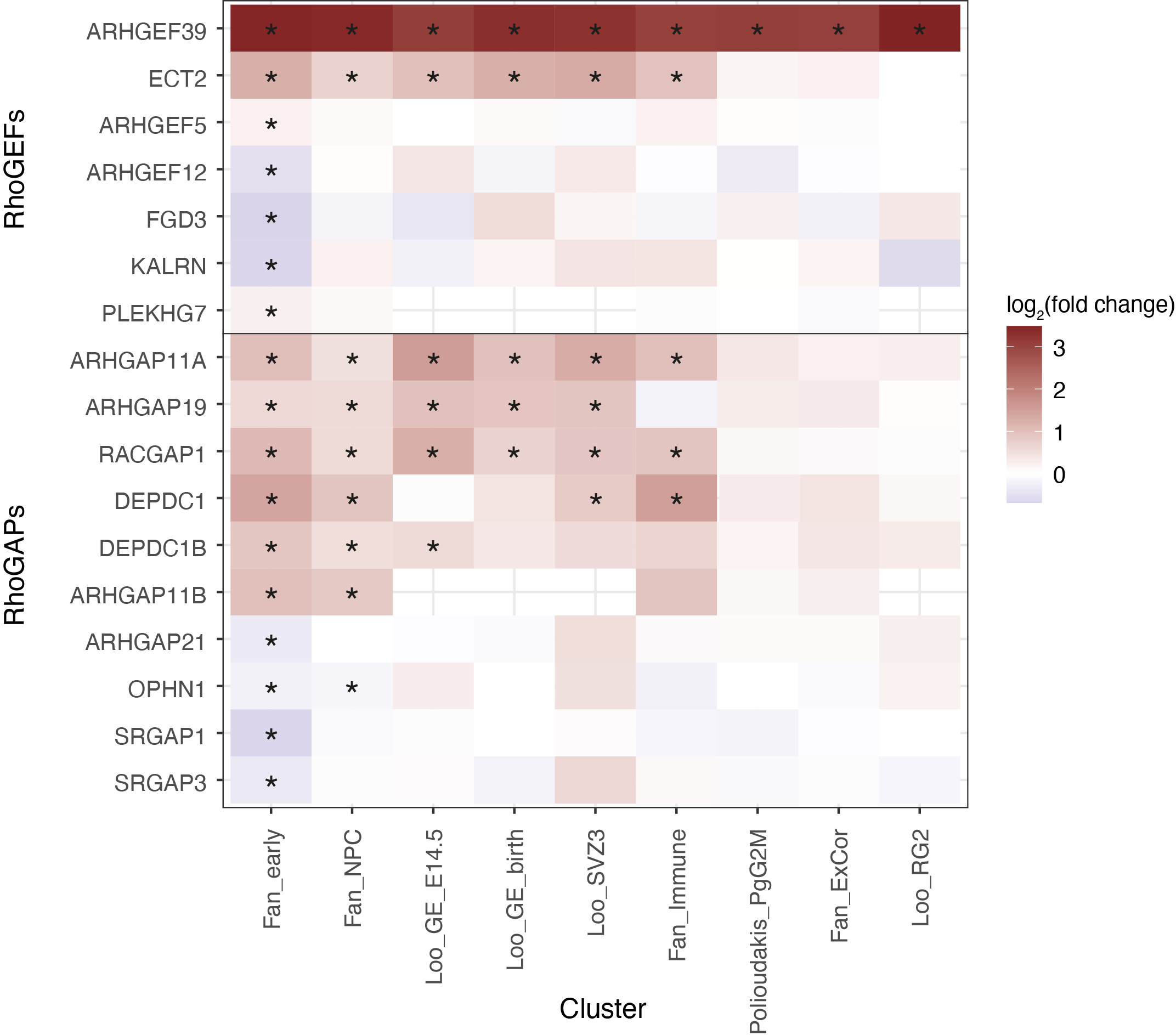


Figure S4. Log_2_ fold changes for differentially expressed RhoGEFs and RhoGAPs.
* indicates FDR-corrected p-value < 0.01 in DEG analysis
